# Multiplexed in-gel microfluidic immunoassays: characterizing protein target loss during reprobing of benzophenone-modified hydrogels

**DOI:** 10.1038/s41598-019-51849-8

**Published:** 2019-10-28

**Authors:** Anjali Gopal, Amy E. Herr

**Affiliations:** 1 Department of Bioengineering, University of California, Berkeley, Berkeley, California 94720 United States; 2 UC Berkeley/UCSF Graduate Program in Bioengineering, University of California, Berkeley, Berkeley, California 94720 United States; 3 Chan Zuckerberg BioHub, San Francisco, California 94158 United States

**Keywords:** Biomaterials, Bioanalytical chemistry, Microfluidics

## Abstract

From whole tissues to single-cell lysate, heterogeneous immunoassays are widely utilized for analysis of protein targets in complex biospecimens. Recently, benzophenone-functionalized hydrogel scaffolds have been used to immobilize target protein for immunoassay detection with fluorescent antibody probes. In benzophenone-functionalized hydrogels, multiplex target detection occurs via serial rounds of chemical stripping (incubation with sodium-dodecyl-sulfate (SDS) and β-mercaptoethanol at 50–60 °C for ≥1 h), followed by reprobing (interrogation with additional antibody probes). Although benzophenone facilitates covalent immobilization of proteins to the hydrogel, we observe 50% immunoassay signal loss of immobilized protein targets during stripping rounds. Here, we identify and characterize signal loss mechanisms during stripping and reprobing. We posit that loss of immobilized target is responsible for ≥50% of immunoassay signal loss, and that target loss is attributable to disruption of protein immobilization by denaturing detergents (SDS) and incubation at elevated temperatures. Furthermore, our study suggests that protein losses under non-denaturing conditions are more sensitive to protein structure (i.e., hydrodynamic radius), than to molecular mass (size). We formulate design guidance for multiplexed in-gel immunoassays, including that low-abundance proteins be immunoprobed first, even when targets are covalently immobilized to the gel. We also recommend careful scrutiny of the order of proteins targets detected via multiple immunoprobing cycles, based on the protein immobilization buffer composition.

## Introduction

Assessing protein-mediated cell-signalling for a wide range of biological and clinical questions (e.g., proliferation^1^, senescence^2^, tumour progression^3^) benefits from bioanalytical techniques developed to interrogate complex cell systems (i.e., cell lysates^4–6^, cell cultures^7–11^, and tissue samples^12,13^). Hydrogels are increasingly used as an immobilization substrate for immunoassays. Hydrogels are biologically inert^14^, offer useful mass transport properties^14^, are ready functionalized with biological and non-biological materials (e.g., extracellular matrix proteins or photoactivatable crosslinkers)^9,10,15^, and are capable of forming either 2D or 3D structures^9,15^. Furthermore, hydrogel-based assays have dramatically improved biological measurement capabilities. For instance, optical-clearing methods (e.g., CLARITY and expansion microscopy) utilize the mass transport and swelling properties of hydrogels to visualize intact brain tissue architecture^12,13,16^. Moreover, covalent chemistries are routinely used to bind cellular material to the hydrogel matrix, especially when rapid, diffusion-driven dilution of solubilized biospecimens will degrade limits-of-detection^12,13,17,18^.

Recently, benzophenone has been utilized as the chemistry of choice to facilitate covalent attachment of biospecimen targets to otherwise inert materials, such as hydrogels. Often, benzophenone is grafted onto a surface or incorporated into a hydrogel matrix such as polyacrylamide (PA)^4,19,20^; subsequent UV irradiation facilitates the formation of benzophenone free radicals that abstract hydrogen atoms from proximal peptide residues, resulting in covalent bond formation between the benzophenone group and nearby protein targets^21^. In some microfluidic devices, this entire process occurs in as little as 45 s^4^. Benzophenone photochemistry is used in a range of bioanalytical research, including the analysis of stem cell differentiation in spatially varying patterns of biomolecules^22^, the development of microfluidic tools to understand enzyme and antibody kinetics^23,24^, and the development of separations to probe isoforms from few numbers of cells^4,5,20^.

In hydrogels functionalized with benzophenone methacrylamide, detection of protein targets adopts standard immunocytochemistry (ICC) or immunohistochemistry (IHC) procedures^4,22^. Specifically, a protein-decorated hydrogel is incubated with primary and secondary antibody probes, and subsequent wash steps remove non-specifically-bound immunoreagents. The secondary antibody probes are most commonly labeled with fluorophores. To read out signal, the hydrogel is imaged with a fluorescence microscope (including confocal and two-photon microscopes) or a laser scanner^4,12,18^. However, detecting multiple protein targets in one specimen (multiplexing) is subject to limitations of fluorescence imaging: in particular, multiplexing is restricted by the standard 4–6 colour channels available in conventional epifluorescence microscopes^25^. Combinatorial post-processing techniques (e.g., spectral unmixing^26^) and fluorophore bleaching or quenching chemistries^27^ have been explored for single-cell ICC and IHC; however, both techniques rely on fluorescently-labeled primary antibodies, which may reduce antibody-antigen binding affinity^28^ and prohibit signal amplification made available by the use of secondary antibody probes for target detection^29^.

An alternate method of multiplex target detection, which has been utilized in some ICC/IHC procedures^30–32^, slab-gel western blots^33^, and in optical clearing assays^12,34^, involves chemical “stripping and reprobing” or “de-staining and reprobing”. Stripping and reprobing chemistries utilize harsh denaturing agents, such as sodium-dodecyl-sulfate (SDS), urea, and/or ß-mercaptoethanol, as well as the addition of heat, to remove immunoreagents from a sample, followed by reprobing of the sample with a new round of immunoreagents^33^. In slab-gel western blotting, proteins adhere onto the PVDF or nitrocellulose membrane via non-covalent interactions; as a result, protein species are denatured and unbound from the membrane upon each stripping cycle. Consequently, standard immunoblotting protocols recommend limiting the number of “stripping and reprobing” cycles to 3–4 rounds^35^.

**Figure 1:**
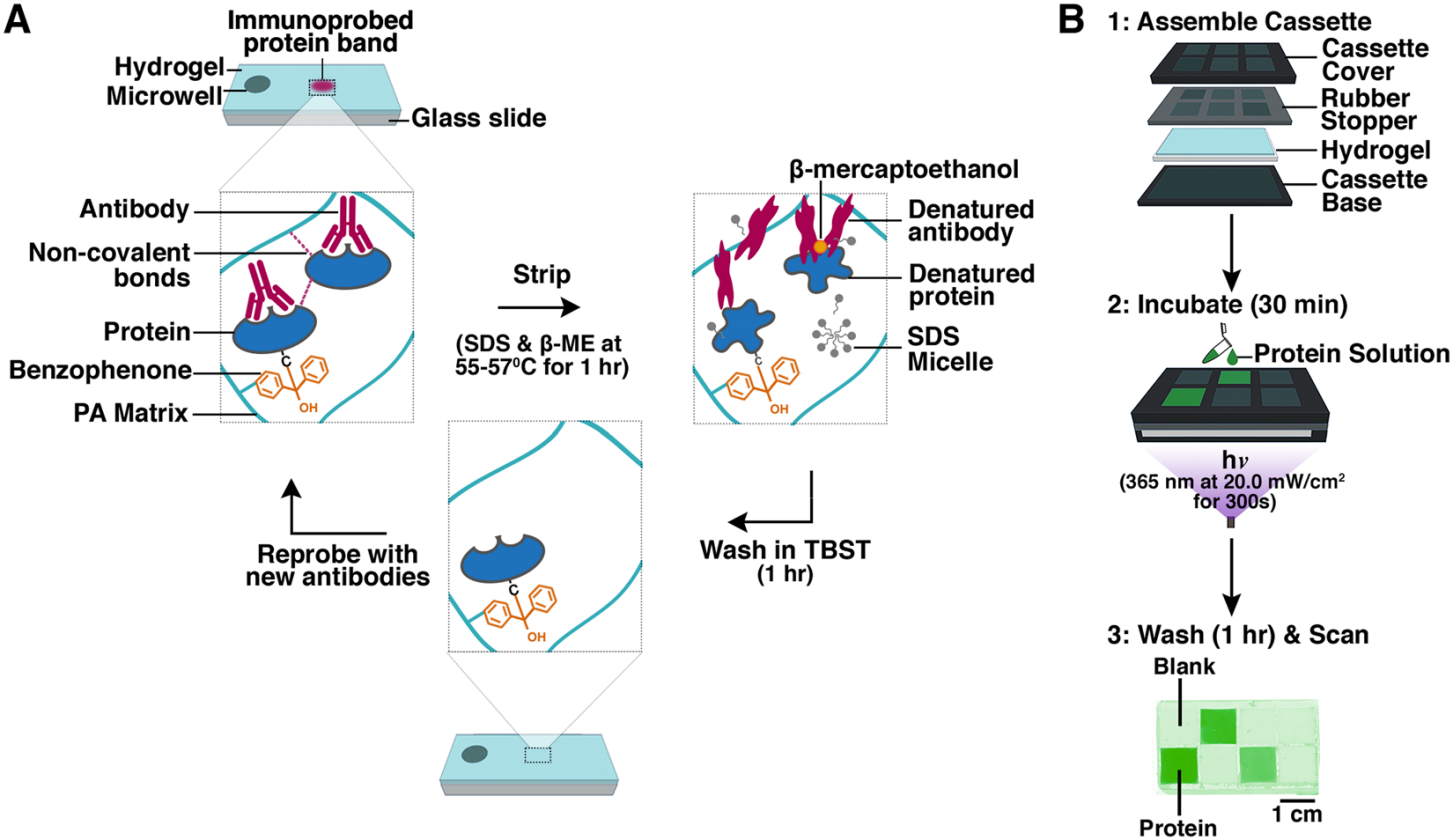
Hypothesized mechanism of immunoassay signal loss in BMPA hydrogels and device fabrication to test hypothesis. (**A**) In BMPA hydrogels, protein targets of interest are covalently bonded to the hydrogel matrix by a benzophenone moiety. However, we hypothesize that some protein targets are immobilized in the gel matrix via non-covalent interactions (e.g., hydrogen bonding, hydrophobic bonding, or van der Waals forces). We hypothesize that the chemical stripping procedure used in multiplexing for hydrogel-based heterogeneous immunoassays denatures these non-covalent interactions, resulting in protein loss from the hydrogel. (**B**) To monitor and quantify protein loss from BMPA hydrogels during chemical stripping & reprobing, we used an ArrayIt Microarray Gasket to create physically isolated 1 cm^2^ arrays in the hydrogel. We loaded purified protein conjugated with fluorescent dye into pre-selected arrays, and filled remaining arrays with buffer. After a 30 min incubation step, gels are exposed to UV irradiation (365 nm, at 20.0 mW/cm^2^) for 300 s, and are subsequently washed and dried. Gels are scanned with a laser microarray scanner to measure fluorescence. We can now test our hypothesis by subjecting these hydrogels to multiple rounds of stripping and reprobing, wherein protein-conjugate fluorescence is used as a proxy for protein concentration, which allows us to monitor protein loss from the hydrogel across serial stripping rounds.

Our group has introduced photoactive hydrogels consisting of benzophenone methacrylamide co-polymerized with polyacrylamide (BMPA hydrogels) as the basis for a suite of electrophoretic protein cytometry (EPC) assays, including size-based electrophoresis, native electrophoresis, and isoelectric focusing, in order to detect proteoforms in single-cell lysate^4–6^. Detection of protein targets occurs by heterogeneous immunoassays^4–6^. At present, we have reported detection of up to twelve sets of individual protein targets from each cell lysate using stripping and reprobing procedures^36^ (see Fig. 1). Furthermore, in a photoactive BMPA hydrogel, we expect minimal loss of immobilized protein targets during multiple stripping and reprobing cycles, as the targets are covalently immobilized to the hydrogel, and the stripping buffer (i.e., SDS, ß-mercaptoethanol, and 50–60 °C temperatures) is not expected to denature C-C covalent bonds (see Fig. 1)^37–39^. However, our observations during stripping and reprobing suggest up to a 50% loss in immunoassay signal upon 12 rounds of reprobing^4^. Whether this immunoassay signal loss is due to immobilized protein loss from the hydrogel or due to other inefficiencies is not yet understood.

Consequently, in this study, we scrutinize the impact of stripping chemistries on protein targets covalently immobilized to BMPA hydrogels via benzophenone. We first develop BMPA hydrogels immobilized with fluorescently labeled proteins. With these hydrogels, we quantify the effect of harsh detergents on protein species upon multiple rounds of stripping. By utilizing fluorescently labeled proteins, we are able to quantify protein retention independent from immunoassay signal. With this system, we: (i) determine the behavior of protein signal upon 29 rounds of stripping, (ii) determine the mechanism of protein loss, and (iii) utilize the acquired knowledge to formulate design guidance for multiplexed, serial in-gel immunoassays.

## Results

Several studies have demonstrated the use of stripping and reprobing chemistries for multiplexing, including reports of 7–10 additional rounds of reprobing in optical clearing assays^34^, up to 10 rounds of additional reprobing in ICC/IHC using specialized immunoreagents linked to quantum dots^32^, and for detection of up to 12 protein targets in EPC^36^. In EPC, the stripping procedure consists of incubating immunoprobed BMPA gels in a cocktail of harsh detergents containing 2% SDS and 0.8% β-mercaptoethanol at 55–57 °C for >1 h^4^. BMPA gels are subsequently washed in 1X Tris-Buffered Saline with Tween-20 (“TBST”) for 1 h to remove residual SDS and β-mercaptoethanol from the hydrogel. Once stripping of the detection antibody is complete, gels can be reprobed to detect additional protein targets (see Fig. 1).

In theory, the stripping procedure in EPC should not disrupt the covalent binding of the protein species to the BMPA matrix, due to the fact that SDS disrupts hydrophobic interactions in proteins^37^ and β-mercaptoethanol disrupts disulfide bridges^38^. Furthermore, exposure to temperatures between 50–60 °C is anticipated to only disrupt additional non-covalent interactions (e.g., hydrogen bonds)^39^. Nevertheless, our observations of the stripping and reprobing phenomena have reported up to a 50% reduction in immunoassay signal while reprobing the same protein target^4^. Thus, as a first step, we sought to investigate whether the observed immunoassay signal loss could be attributable to loss of protein target from the BMPA hydrogel, due to denaturation of non-covalent protein-hydrogel interactions (e.g., hydrogen bonding^40,41^, hydrophobic interactions^42^, or van der Waals forces^42^) by application of detergents such as SDS and heat (see Fig. 1).

### Development of BMPA hydrogels with immobilized protein

To understand the mechanism of protein loss during stripping of BMPA hydrogels, we first sought to quantify protein signal in these hydrogels after several rounds of incubation in stripping buffer, in a manner that was independent of immunoassay signal. In order to create a model system, we incubated fluorescently labeled purified proteins with the BMPA hydrogels. We did not use naturally fluorescent proteins (e.g., GFP), which have fluorescence that is often dependent on the secondary structure of chromophore-stabilizing polypeptide chains. For instance, GFP is highly susceptible to fluorescence loss by exposure to denaturants (SDS) at low pH (<6.5)^43^. Instead, in our system, we labeled the target proteins with an Alexa Fluor dye, purported to be among the brightest and most photostable commercially available protein dyes^44^. Furthermore, previous studies of immobilized proteins in BMPA hydrogels have reported that micro-to-nanomolar quantities of protein conjugates labeled with Alexa Fluor dyes have fluorescence that linearly increases with protein molarity^24^. As a result, we measure protein-conjugate fluorescence intensity as a proxy for protein concentration in the hydrogel. Our protein target is Bovine Pancreatic Ribonuclease A (RNase), labeled in-house with Alexa Fluor 488 (RNase-488). Thermocycling experiments confirmed that the protein/dye conjugate does not lose fluorescence upon temperature cycling, as long as the temperature during the endpoint measurement is held constant (see Supplementary Fig. S1).

To create hydrogels decorated with immobilized purified protein, we fabricated BMPA gels as previously described^4^, and used a gasket system (ArrayIt Microarray Gasket, ArrayIt Corporation, Sunnyvale, CA) to physically isolate regions of the BMPA gel and selectively dose these regions with protein (see Fig. 1B). Furthermore, in each row of wells, we ensured that one of the two wells was filled with buffer to create “blank” regions for subsequent determination of gel background fluorescence. The purified protein solution was incubated for 30 min, which corresponds to the 4τ diffusion time for large (i.e., 150 kDa) proteins into 30–40 μm thick BMPA gels^17,45^. After incubation in protein solution, gels were irradiated with UV light for 300 s to enable covalent bond formation between the benzophenone moieties and the purified protein species. The final gel consisted of three immobilized protein regions, which contained protein signal, surrounded by regions of background signal. With the development of BMPA hydrogels with immobilized protein, we then proceeded to subject these hydrogels to serial incubation cycles in stripping buffer in order to quantify protein loss after multiple stripping rounds.

### Quantifying protein-conjugate fluorescence after multiple stripping cycles

**Figure 2:**
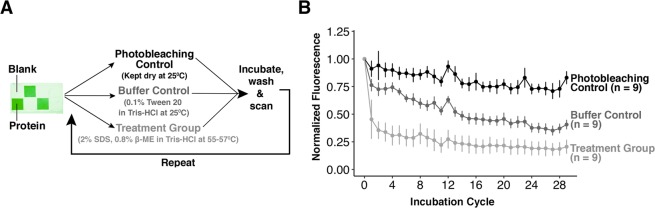
Monitoring loss of immobilized protein target from BMPA hydrogels during chemical stripping. (**A**) 9 sets of BMPA hydrogels with immobilized protein were fabricated. These hydrogels were split into 3 triplicate groups consisting of the photobleaching control, the buffer control, and the treatment group. Each group contains n = 9 immobilized protein regions. (**B**) After each round of incubation, the mean integrated intensity of each immobilized protein region was analyzed. 5.49 mm × 5.04 mm ROIs were defined in each immobilized protein region, from which fluorescence intensity values were summed and normalized to the starting fluorescence intensity. The treatment group, which is incubated in stripping buffer, demonstrates substantial (>50%) loss in protein signal in the first 4 rounds of stripping. By contrast, the buffer control experiments demonstrate a steady decrease in fluorescence (~5% per round) until round 16, at which point the rate of signal loss decreases.

In order to quantify protein loss after serial stripping cycles, (see Fig. 2A), we developed a quantitative method to compare before-and-after fluorescence intensities of hydrogel micrographs after each round of incubation in stripping buffer. For each experiment, hydrogels were incubated in stripping buffer, or in one of two control conditions, for 1 h. With the exception of photobleaching controls, all gels were also washed in 1X TBST for 1 h. Gels were subsequently rinsed with DI water, dried under an N_2_ stream, and scanned with a laser microarray scanner.

Once incubation experiments were complete, we defined the areas in each immobilized protein region from which protein signal would be collected for downstream analysis (see Supplementary Fig. S2). First, all fluorescence micrographs for a single gel were feature-matched and compiled into a TIFF stack using MATLAB’s implementation of the Speeded Up Robust Features (‘SURF’) algorithm. Specifically, SURF extracts and utilizes local features of each micrograph^46^ to align micrographs obtained from subsequent stripping cycles to the initial micrograph. As a result, we are able to perform pixel-by-pixel fluorescence intensity comparisons between micrographs obtained in sequential stripping rounds. Once all micrographs were stacked and aligned, we identified a 5.49 mm × 5.04 mm region-of-interest (ROI) in each immobilized protein region.

We next sought to quantify the protein fluorescence from these ROIs across multiple stripping cycles. In order to quantify the fluorescence, we fit the distribution of pixel intensities in each ROI to a Gaussian distribution. To exclude effects from outlier pixels in each ROI (i.e., “hot” pixels with high intensity, or dark pixels with an intensity close to 0), we calculated the median intensity value of all pixels and re-scaled any pixel values that were greater than 4 standard deviations from the mean (corresponding to <1% of the total distribution of pixels) to the median pixel value. We then summed all pixel values from the resulting distribution to calculate the total fluorescence intensity of each ROI. Furthermore, we performed background subtraction by identifying an identically sized ROI in a background region adjacent to each immobilized protein region, and subsequently quantified the fluorescence intensities from these background ROIs in the same manner described for protein ROIs. Finally, in order to have a direct comparison of protein fluorescence before and after stripping, we normalized the summed intensity value of each ROI from subsequent stripping cycles to the summed intensity value of the ROI obtained from the starting micrograph.

We posit that the formulation of a protein-conjugate fluorescence monitoring system across multiple stripping rounds provides a robust and quantifiable method of tracking the response of multiple types of proteins across multiple hydrogel systems (i.e., with different immobilization chemistries), in addition to BMPA hydrogels.

### Investigating Protein-Conjugate Fluorescence after Sequential Stripping Rounds

We next scrutinized the hypothesis that the observed ~50% decrease in immunoassay signal intensity after 12 rounds of immunoprobing^4^ arises from loss of non-covalently-bound proteins from the BMPA gel. Although we anticipate that most immobilized protein species are covalently bound to the benzophenone moiety in the gel, a subset of protein molecules may be bound via non-covalent interactions (e.g., hydrogen bonding^40,41^, hydrophobic interactions^42^, van der Waals forces^42^), either to the hydrogel matrix or to other covalently bound proteins in the gel. We hypothesized that non-covalent interactions of proteins not immobilized to benzophenone would be disrupted by harsh, ionic detergents (SDS) and incubation in elevated temperatures (55–57 °C incubation for 1 h). As a corollary, we further hypothesized that the majority of the protein loss would occur in the first 2–3 rounds of stripping, after which any remaining protein species would be covalently bound to the hydrogel matrix. Since we utilize protein fluorescence as a proxy for protein concentration, we anticipated a decrease in fluorescence intensity during the first 2–3 rounds of stripping, followed by a plateau in fluorescence.

Our results indicate that the first 5 stripping cycles see the majority of protein signal loss (>50% signal loss) from the treatment group (see Fig. 2B). We observe gradual signal loss from the treatment group after round 5, which we attribute to repeated photobleaching of target by the laser microarray scanner. Similar levels of normalized signal are lost during rounds 11–29 for both the treatment group and the photobleaching controls, after normalization to round 11 (see Supplementary Fig. S3). To assess the similarity between the photobleaching controls and the stripping samples in rounds 11–22, we performed Mann-Whitney U-tests on the residual signal intensity in each round (see Supplemental Table S1). The Mann-Whitney U-tests demonstrate that either (1) the p-value is greater than 0.05, indicating no significant differences between the two groups, or that (2) for groups where the p-value is <0.05, the treatment group has greater mean normalized fluorescence intensity (i.e., lower signal loss) than the photobleaching control group (see Supplemental Table S1). Thus, between rounds 11–22, the signal loss from the treatment group during these cycles is indistinguishable from photobleaching effects. As an important aside, we note that the spike in protein-conjugate signal intensity observed during cycle 12 is attributable to scanner variation, owing to a concomitant increase in signal intensity across all control conditions.

Furthermore, minor protein loss observed across all conditions at round 24 is attributed to a two-month delay between scanning round 23 and round 24 for all groups. Nevertheless, when we normalize the fluorescence signal intensity to round 23, we once again observe indistinguishable sample loss between the photobleaching control group and the treatment group during rounds 24–29 (see Supplementary Fig. S4, and see Supplemental Table S2 for p-values of Mann-Whitney U-Tests).

Moreover, the majority of the signal loss for the buffer controls occurrs during the first 16 cycles. We hypothesize that the difference in final protein-conjugate signal intensities between the treatment group and the buffer controls arises from differences in buffer composition: although both conditions lead to protein denaturation and disruption of protein-protein or protein-hydrogel interactions, the stripping buffer contains SDS and is heated to 55–57 °C. By contrast, the detergent used in the buffer controls is Tween-20, which is a milder, non-ionic detergent that does not disrupt protein-protein or protein-hydrogel interactions as strongly; and moreover, the buffer controls are incubated at room temperature (25 °C), which also results in less protein denaturation. By extension, we would hypothesize that harsher stripping conditions, such as those used in the CLARITY assay (consisting of stripping with 4% SDS at 60 °C for approximately 12 h)^12^ would likely result in greater protein losses during the first few rounds of stripping, and as a result, a corresponding plateau in signal loss during earlier stripping rounds.

**Figure 3:**
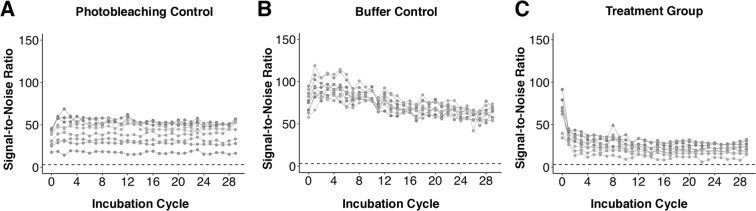
Monitoring SNR of immobilized protein targets from BMPA hydrogels during chemical stripping. (**A**–**C**) SNRs of the photobleaching controls, the buffer controls, and the treatment group, respectively. The SNRs of the photobleaching control gels demonstrate minimal fluctuation over 29 incubation cycles, whereas the SNRs of the buffer control gels demonstrate a steady decrease for the first 14–16 cycles, followed by a plateau. The SNRs of the treatment group demonstrate a dramatic decrease during the first 3–5 rounds of stripping, followed by a plateau for the remaining rounds. For all plots, each trace represents the SNR from one ROI in a BMPA hydrogel.

Additionally, we sought to understand the impact of the stripping buffer on the signal-to-noise ratios (SNR) of the BMPA hydrogels with immobilized protein (see Fig. 3, where each trace represents a single ROI). The SNR is characterized as the ratio between the mean signal intensity and the background noise. A SNR > 3 is a threshold for exceeding the limit-of-detection (LOD) of an assay. Since there is some variation in the starting intensity for every immobilized protein ROI, due to variation in protein entry into local regions of the BMPA hydrogel, we tracked the SNR of each ROI individually over multiple stripping cycles.

Our results demonstrate that the trends in SNR are similar to the trends in normalized fluorescence intensity values for each sample condition: the photobleaching control has minimal fluctuation in the SNR values, corresponding to photobleaching or measurement variation; the buffer control samples have a steady decrease in SNR until rounds 14–16, after which the values have minimal variation; and the treatment group has a sharp decrease in SNR, followed by a plateau. Moreover, for the treatment group, the greatest change in SNR occurs in the first 3–5 rounds, which is once again in agreement with the trend observed for normalized fluorescence intensity values.

Finally, by observing the ROI of the immobilized protein region with the lowest SNR value in the treatment group (an SNR of 6.35 at round 22), and by comparing this to the SNR value of the same ROI at round 4 (SNR ~ 12 for the same ROI at round 4), we can draw additional conclusions about the LOD our system: specifically, our results suggest that if the SNR of an observed ROI is above 12 by round 4, then the SNR of the same ROI will continue to remain above 3 (i.e., the LOD) for the remainder of the 29 stripping cycles.

In accordance with western blotting, our observations suggest that low-abundance proteins should be immunoprobed first despite utilization of covalent chemistries for protein immobilization^33,47^, as our analysis indicates that any proteins that remain in the gel after the fourth stripping cycle will continue to remain in the gel for a minimum of 25 additional stripping cycles.

### Investigating the mechanism of protein loss from stripping rounds

We next sought to investigate the primary mechanism of protein loss from BMPA hydrogels after sequential stripping rounds. Recall, we hypothesized that the main mechanism of protein loss was disruption of non-covalent interactions between the protein and the hydrogel matrix, or from protein-protein species^40–42^. Moreover, we hypothesized that the primary contributors to protein loss are SDS and heat, as both SDS and heat disrupt hydrophobic bonds and van der Waals interactions, and heat also disrupts hydrogen bonding^37,39^. By contrast, β-mercaptoethanol (the other main component of stripping buffer) primarily denatures disulfide bridges^38^. Although disulfide bridges are a critical component of immunoreagents, we do not anticipate that BMPA hydrogels with immobilized RNase-488 contain significant intermolecular disulfide bridges. Therefore, we hypothesized that the contribution of protein loss from β-mercaptoethanol would be minimal.

**Figure 4:**
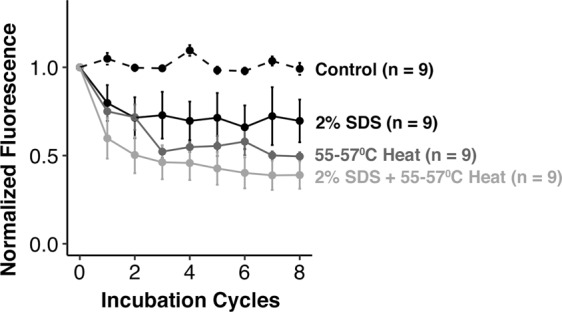
SDS and heat are primary contributors to protein loss during the stripping process. Four sets of triplicate BMPA hydrogels with immobilized RNase-488 were exposed to (i) photobleaching only (control), (ii) just detergent (2% SDS), (iii) just heat (55–57 °C temperatures), or (iv) detergent and heat (2% SDS + 55–57 °C heat). At round 8, the combination of the detergent and heat condition had the greatest fluorescence intensity loss (61.1% ± 7.7%).

We created four sets of triplicates of BMPA hydrogels immobilized with RNase-488 (12 gels total), to examine the loss of protein signal upon exposure to only 2% SDS, only heat, and a combination of 2% SDS and heat. Our results indicate that, after 8 incubation cycles, the SDS-only treatment led to a 30.4% ± 12.1% average decrease in fluorescence intensity, the heat-only treatment led to a 50.5% ± 5.7% average decrease in fluorescence intensity, and the combination of the two conditions led to an average 61.1% ± 7.7% average decrease in fluorescence intensity (see Fig. 4). By contrast, the initial RNase-488 stripping experiments described in Fig. 2, which used all stripping buffer components (SDS, β-mercaptoethanol, and heat), had an average 67.9% ± 9.0% decrease in fluorescence intensity at round 8. A Mann-Whitney U-test indicated that the difference in fluorescence decrease between the SDS/heat-only experimental condition and the “full stripping buffer” experimental condition is not statistically significant (p-value = 0.07701); as a result, our hypothesis that the majority of protein signal loss occurs due to SDS and heat is not falsified. Furthermore, a Mann-Whitney U-test also reveals that the difference between the 2% SDS control (average signal loss of 30.4% ± 12.1% at round 8) and the TBST buffer control performed in our initial experiment (average signal loss of 40.2% ± 4.5% at round 8) is also not statistically significant (p-value = 0.1359), which indicates that TBST and SDS do not induce significantly different protein losses upon similar incubation timescales.

Based on these results, we recommend stripping buffer formulations that provide stringent removal of immunoreagents but without SDS or elevated temperatures. We anticipate that alternative formulations can be sought that retain immobilized target on BMPA hydrogels for a greater number of immunoprobing rounds, thus facilitating detection of lower-abundance proteins.

### Understanding the role of molecular mass in protein loss

As a next step, we sought to understand the behaviour of protein loss with protein species of different molecular mass. Previous studies have reported that the photocapture efficiency of benzophenone varies with the molecular mass of each immobilized target protein species. Photocapture efficiencies of 75.2%, 93.1% and 97.5% were reported for trypsin inhibitor (TI, 20.1 kDa), ovalbumin (OVA, 42.7 kDa), and beta-galactosidase (116 kDa) respectively^17^. As a result, we hypothesized that the final protein fluorescence signal plateau would also vary in a size-dependent manner, with the smallest proteins experiencing the largest loss (corresponding to the lowest photocapture efficiency), and the largest proteins experiencing the smallest loss (corresponding to the largest photocapture efficiency).

We fabricated BMPA hydrogels with immobilized protein, consisting of either fluorescently labeled OVA or TI, and performed stripping experiments identical to those performed with RNase. Once again, each protein species had triplicate gels that functioned as either the photobleaching control or as the treatment group.

**Figure 5:**
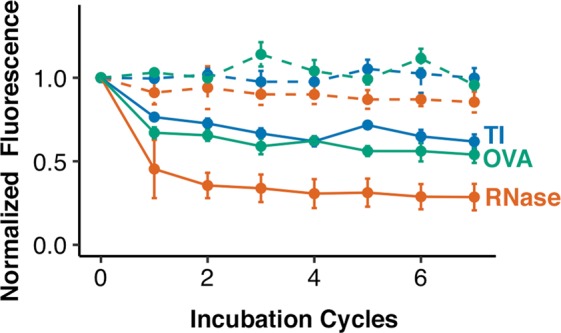
Protein size is not a substantial contributor to protein loss in non-denaturing photocapture conditions. Triplicate BMPA hydrogels with immobilized protein were created for each of OVA (42.7 kDa), TI (20.1 kDa), and RNase (13.7 kDa). Dotted lines represent photobleaching controls, whereas solid lines represent treatment groups. At round 7, the protein losses were 46.0% ± 5.0%, 38.2% ± 4.2%, and 71.4% ± 7.9% for OVA, TI, and RNase, respectively, indicating minimal size-based correlation.

Our results suggest that protein molecular mass is not a significant factor in protein loss (see Fig. 5). Although RNase, the smallest protein, has the greatest amount of protein loss, the trends in protein loss do not correlate with the molecular masses of the other two proteins, OVA and TI (see Table 1).

**Table 1:** Physicochemical Properties of Proteins Immobilized in BMPA Hydrogels and their Respective Signal Losses upon 7 Rounds of Stripping.

Protein	Molecular Mass	Hydrodynamic Radius	Signal Loss
RNase	13.7 kDa	1.73 nm^49^	71.4% ± 7.9%
OVA	42.7 kDa	2.65 nm^49^	46.0% ± 5.0%
TI	20.1 kDa	3.4 nm^48^	38.2% ± 4.2%

To reconcile this discrepancy, we first note that proteins immobilized in the BMPA hydrogels were incubated in a solution of 2% BSA/TBST prior to UV photocapture via benzophenone. However, previous studies of benzophenone photocapture efficiency utilized SDS to denature proteins prior to UV irradiation^17^. We expect that when proteins are denatured, benzophenone moieties have access to most of the amino acid residues in the peptide chain, from which hydrogen atoms can be abstracted for photocapture^21^. Although benzophenone does demonstrate residue-specific preferences during photocapture^21^, we can nevertheless assume that, to a first approximation, photocapture efficiency scales with the amount of abstractable hydrogen atoms that are accessible to the benzophenone moiety. We further know that all amino acid residues in a peptide chain possess at least one abstractable hydrogen group (i.e., at minimum, the alpha carbon)^21^. Thus, under denaturing conditions, we expect that photocapture efficiencies scale with the size of the protein.

However, in non-denaturing conditions, we expect that not all amino acid residues would be accessible by benzophenone (e.g., residues that are buried inside hydrophobic pockets of an amino acid chain). We investigated whether the hydrodynamic radius of the protein might be a proxy for the number of amino acids accessible to benzophenone, since globular proteins with larger radii would also have larger surface areas, and thus, more surface accessible amino acids. Previous studies estimate that the hydrodynamic radius of RNase, TI, and OVA are approximately 1.73 nm^49^, 3.4 nm^48^, and 2.65 nm^49^ respectively; the rank ordering of these radii matches the rank ordering of signal loss observed for these protein targets (see Table 1). Thus, in non-denaturing conditions, we anticipate that protein retention is more correlated with hydrodynamic radius than protein molecular mass.

Based on these results, we recommend both (i) careful choice of immobilization buffer during photocapture with benzophenone and (ii) anticipation of either size-based, or hydrodynamic radius-based, signal loss for protein target, depending on the use of denaturing or non-denaturing buffer conditions.

### Comparing protein-conjugate fluorescence loss to immunoprobed fluorescence loss

Finally, in order to evaluate the contribution of protein loss to immunoassay signal loss, we next created two sets of triplicates (photobleaching control and treatment group) of BMPA hydrogels immobilized with RNase-488 and subjected these groups to serial rounds of stripping, followed by immunoprobing. We immobilized a smaller concentration of RNase-488 (125 nM in-solution concentration of RNase-488) with a higher degree of labeling of Alexa Fluor 488 (DOL = 1.23) in order to ensure that the concentrations of immunoprobing reagents, which were utilized at 0.1 mg/mL, would be in excess of the in-gel RNase-488 concentration, while also ensuring that we would be able to sufficiently visualize our RNase-488 conjugates during multiple stripping cycles. We stripped, and reprobed, our RNAse-488 gels for a total of 6 times.

**Figure 6:**
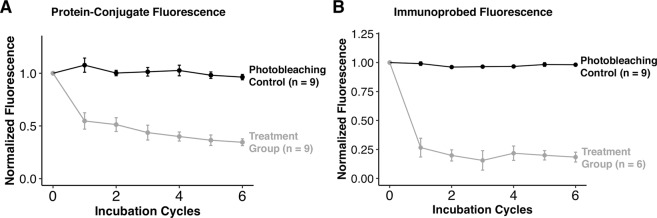
Comparison of Fluorescence Loss Between Protein-Conjugate and Immunoreagents during Serial Stripping and Reprobing Cycles. (**A**) Fluorescence loss of protein-conjugates in three BMPA hydrogels immobilized with RNase-488 (DOL = 1.3). Round 0 corresponds to the RNase-488 fluorescence after the initial immunoprobing round. (**B**) Fluorescence loss of immunoreagents in BMPA hydrogels immobilized with RNase-488 and immunoprobed with primary (Rb anti-RNase) and fluorescent secondary (Gt anti-Rb conjugated to Alexa Fluor 633) antibodies. Each incubation cycle following round 0 corresponds to one round of stripping, followed by immediate reprobing with new immunoreagents.

We quantified the fluorescence from our immunoprobed hydrogels in a manner identical to the quantification performed with our protein-conjugate hydrogels. Furthermore, we performed background subtraction from regions absent protein-conjugate (but incubated in immunoreagents) to account for any confounding signal from blank hydrogel regions. Our results demonstrate that the protein-conjugate fluorescence (see Fig. 6A) follows the same trend observed in our initial experiments of RNase-488 fluorescence loss after multiple stripping rounds as depicted in Fig. 2. In our stripping and reprobing experiments, the treatment group demonstrated a loss of 65.5% ± 3.2% after 6 rounds (n = 9), with the majority of the protein loss occurring in the first 1–3 rounds. A Mann-Whitney U-test indicated no significant difference (p-value = 0.077) between the observed fluorescence loss of the treatment group in the current experiment, and the observed fluorescence loss of the treatment group in the initial stripping experiment after 6 rounds of stripping (71.2% ± 7.5%, n = 9).

Finally, we investigated loss of immunoassay signal after multiple reprobing rounds of our RNase-488 protein conjugate. We observed the majority of immunoassay signal loss in the first round; by round 6, the treatment group demonstrated 81.6 ± 4.25% signal loss (n = 6), which is substantially higher than the protein-conjugate fluorescence loss (see Fig. 6B**)**. This result suggests that protein conjugate loss may be responsible for the majority, but not all, of immunoassay signal loss.

As an important aside, we observed spatially non-uniform probing results in one of the three gels in the treatment group, which we attribute to our reagent-sparing probing process (antibody concentration at 0.1 mg/mL; application of ~40 μL solution using a glass plate^50^). We did not include results from the questionable gel in the proceeding analysis (see Supplementary Fig. S5, Supplemental Table S3, Supplementary Fig. S6**)**.

We hypothesize that any remaining immunoassay signal loss that is not attributable to protein loss may be caused either by (1) epitope masking, due to the exposure of protein target to denaturing detergents, such as SDS, during the stripping procedure, or (2) hindered immunoreagent entry into the BMPA gel during successive immunoassay probing rounds. We put substantial weight on the second hypothesis because we have observed residual immunoreagent fluorescence after each stripping round, which increases after every reprobing round (see Supplementary Fig. S7). We suspect that this increase is due to entropic trapping of immunoreagents among the pores of the PA gel; although estimates of the antibody hydrodynamic radius (~5 nm)^51^ are much smaller than estimates of the PA pore size for 7–8%T gels (~50–90 nm whilst utilizing a 3–4% bis-acrylamide crosslinker concentration^52^), previous studies have demonstrated that a 1:10 difference in particle-to-pore size is sufficient for entropic trapping of solutes in a hydrogel matrix^53^. We can expect that entrapment of antibodies in the PA gel would hinder further immunoreagent entry, owing to inaccessible pores through which new immunoreagents can no longer migrate, which would ultimately reduce antibody-to-analyte complex formation, and thus, result in additional signal loss.

## Discussion

As a basis for design of multiplexed in-gel immunoassays, we scrutinized the effects of harsh denaturing detergents on protein targets covalently immobilized to a BMPA hydrogel. The majority of immunoassay signal loss is attributed to loss of non-covalently-bound proteins from the gel matrix, and occurs during the first 5 rounds of stripping. By tracking fluorescence signal from protein-fluorophore conjugates, we determine that protein loss plateaus starting from the 11^th^ stripping round (if not earlier) and is maintained through the 29th stripping round. Our results suggest that, despite utilizing covalent chemistries to immobilize proteins to BMPA hydrogels, low-abundance protein targets should be immunoprobed first, as in accordance with slab-gel western blotting best practices.

Moreover, we surmise that the main contributors to protein loss are (i) the SDS component of the stripping buffer and (ii) the elevated temperature used during the stripping procedure. The working conclusions suggest that the majority of protein loss is from proteins that are non-covalently bound to the hydrogel. We posit that the investigation of stripping buffers that do not use SDS or heat, but nonetheless remove the majority of immunoprobes, may facilitate lower protein loss during successive immunoprobing cycles.

We further conclude that the molecular mass of protein targets is not a significant contributor to protein loss during non-denaturing photocapture conditions, as differently sized proteins (i.e., RNase at 13.7 kDa, TI at 20.1 kDa, and OVA at 42.7 kDa) demonstrated little size-based correlation in their respective protein losses (71.4% ± 7.9%, 38.2% ± 4.2%, and 46.0% ± 5.0%, respectively). In non-denaturing photocapture conditions, we hypothesize that hydrodynamic radius may be a better correlate for protein loss. Based on these results, we recommend choosing the order of protein target detection in successive immunoprobing cycles not only based on protein abundance, but also based on the key physicochemical properties (e.g., molecular mass or hydrodynamic radius) that may modulate protein retention in different immobilization buffers (denaturing vs non-denaturing).

Our results are consistent with one of the key reported mechanisms for interaction between proteins and hydrogels: hydrogels routinely interact with proteins through non-covalent interactions, including hydrogen bonding^40,41^, electrostatic interactions^42^, and van der Waals forces^42^. Furthermore, protein adsorption through non-covalent interactions facilitates incorporation of biomolecules onto hydrogel scaffolds for tissue engineering. These studies, coupled with our results, suggest that non-covalent interactions can modulate protein retention across a wide range of hydrogels^54^. Furthermore, the “gradual release” of proteins from the BMPA hydrogel system is similar to the controlled release of drugs or small molecules from modified PA hydrogels, some of which demonstrate release of ~56% and ~77% of their payload over the course of 24 and 75 h respectively^55^.

In benchmarking this system against other multiplexed immunoassays, we note that most studies assess antigenic recovery (i.e., presence or absence of protein target signal)^34^. Fewer studies quantify the amount of signal present between successive immunoprobing rounds, when serial multiplexing strategies are used. Nevertheless, previous studies relating antigen losses from surfaces or membranes (e.g., nitrocellulose, nylon, or PVDF) report appreciable antigen signal retention for 3–6 rounds of immunoprobing with similar stripping buffers (i.e., SDS, ß-mercaptoethanol, and heat)^33^. Alternative stripping buffer formulations, including those involving glycine HCl, have reported antigenic detection for up to 21 rounds of protein targets on PVDF membranes^47^. The fact that BMPA hydrogels can extend stripping and reprobing cycles to 29 rounds without alternative stripping buffer formulations suggests that (1) BMPA hydrogels retain more antigen than membranes during successive stripping rounds, and that (2) alternate stripping buffer formulations may support even more antigen retention per round when used in conjunction with BMPA hydrogels.

Looking forward, we envision that optimizing assay chemistries may decrease protein losses during the first few rounds of stripping, enabling detection of low abundance proteins for more reprobing cycles. For instance, we are interested in exploring the potential of quenching and reprobing chemistries with heterogeneous immunoassays in hydrogels to reduce protein loss during the first few reprobing cycles^27^. We additionally surmise that further understanding of entropic trapping of antibodies in hydrogels during immunoprobing may provide additional clues as to the mechanism of immunoassay signal loss during stripping and reprobing rounds.

We anticipate that the results of this study will be broadly applicable to protein-hydrogel bioanalytical tools, and may lead to greater adoption of the stripping and reprobing method for increased multiplexing.

## Methods

### Reagents

30% Acrylamide/bis-acrylamide (29:1) (A3574), N,N,N’,N’-Tetramethylethylenediamine (T9281), ammonium persulfate (A3678), bovine serum albumin (A9418), sodium dodecyl sulfate (L4509), 2-mercaptoethanol (M3148), hydroxylamine hydrochloride (379921), trypsin inhibitor from soybean (65035), ribonuclease A from bovine pancreas (R5500), and albumin from chicken egg white (A5503) were purchased from Millipore Sigma. Tris-HCl, pH 6.8 (T1568) and Tris-HCl pH 8.8 (T1588) was purchased from Teknova Inc. Phosphate-buffered saline (10X PBS Corning, 45001) was purchased from VWR. N-[3-[(3-Benzophenyl)-formamido] propyl] methacrylamide was synthesized by PharmAgra Laboratories. Gibco Phosphate-buffered saline (1X PBS, 10010023), Alexa Fluor 488 Microscale Protein Labeling Kit (A30006), Alexa Fluor 488 TFP ester (A37570), and Alexa Fluor 633 goat anti-rabbit secondary antibody (A21071) were purchased from ThermoFisher Scientific. Bio-Gel P-6 Fine Resin (1504134) was purchased from Bio-Rad Laboratories. Nanosep MF Centrifugal Devices (0.2 μm, ODM02C34) was purchased from Pall Laboratories. Trisbuffered saline with Tween-20 (TBST-10×, 9997 S) was purchased from Cell Signaling Technology. Ribonuclease A primary antibody (100 μL, NBP1-69256) was purchased from Novus Biologicals. Deionized water (18.2 MΩ) was obtained from an Ultrapure Millipore filtration system. ArrayIt Microarray Gaskets (AHC1X16) were purchased from ArrayIt Corporation. Borosilicate glass plates were purchased from McMaster-Carr (8476K17). Diamond-tipped scribes were purchased from Amazon (SCB-431.00).

### Protein labeling

Trypsin inhibitor from soybean (TI), ribonuclease A from bovine pancreas (RNase), and albumin from chicken egg white (OVA) were labeled in-house using the described protocol in the Alexa Fluor 488 Microscale Protein Labeling Kit. With the exception of reprobing experiments, molar ratios of dye:protein of 60, 19, and 0.67 were used for OVA, TI, and RNase, respectively, at the recommended protein concentration of 1 mg/mL, resulting in degrees-of-labeling of 3.1, 1.76, and 0.10 fluorophores per molecule of protein, respectively. For experiments that involved reprobing of RNase, a dye:protein molar ratio of 5 was used, resulting in a degree-of-labeling of 1.23. Finally, for batch OVA and TI labeling reactions, 1/10th volume of 1.5 M hydroxylamine-hydrochloride was used to stop the labeling reaction after 15 min of incubation.

### Fabrication of BMPA hydrogels with lmmobilized protein

600 μL microcentrifuge tubes were blocked overnight with 10% BSA in 1X TBST. 7%T BMPA hydrogels were fabricated as previously described on silanized glass slides^4^. Prior to fabrication, glass slides were vertically scored with a diamond scribe 1.5” down the middle. After fabrication, BMPA gels were briefly incubated in 1X TBST and loaded into the ArrayIt Microarray gasket system (gel-side up). 80 μL of 1X TBST was loaded into each well. To load one-half of the BMPA gel with purified protein solution, 270 μL of 2.1 μM protein solution was created in a BSA-blocked microcentrifuge tube. TBST was aspirated out of 3 microwells of the ArrayIt Gakset, and each empty well was loaded with 80 μL of purified protein solution. The same procedure was repeated for the other half of the BMPA gel. BMPA gels were incubated with protein solution for 30 min, and then exposed to collimated UV light under a mercury arc lamp (~20 mW/cm^2^ at 365 nm, Optical Associates, Inc.) for 300 s. The gasket fixture was then disassembled, and BMPA gels were rinsed in 1X TBST overnight. After an overnight wash, BMPA gels were dried under an N_2_ air stream, and broken in half, producing two half-gels. Half-gels were analyzed with a laser microarray scanner (Genepix 4300 A, Molecular Devices) to measure the resulting fluorescence profiles. Micrographs of gels displayed in figures are false-colored and contrast-enhanced; however, quantitation of gels are performed using raw pixel intensity values.

### Stripping experiments

Harsh stripping buffer was made with 62.5 mM Tris-HCl (pH 6.8), 2% SDS, and 0.8% β-mercaptoethanol. Wash buffer consisted of 1X TBST. For initial stripping experiments, BMPA gels with immobilized RNase-Alexa Fluor 488 (RNase-488) conjugates were created in triplicates for 3 experimental conditions: the photobleaching control, the buffer control, and the treatment group (see Fig. 2A). Photobleaching control gels were kept dry during the duration of the experiment, but scanned with the remaining gels at the end of each stripping cycle. Buffer control gels were incubated in 1X TBST at room temperature (25 °C) for 1 h. Treatment group gels were incubated in stripping buffer at 55–57 °C for 1 h. Both buffer control and treatment group gels were subsequently washed in 1X TBST for 1 h, rinsed with DI water, and dried under a N_2_ stream. All gels were scanned with a laser microarray scanner to track changes in fluorescence profiles.

### Stripping mechanism experiments

To isolate components of stripping buffer that may be contributing to signal loss, buffers consisting solely of 62.5 mM Tris-HCl (pH 6.8), and solely of 62.5 mM Tris-HCl and 2% SDS were also created. Gels were created in triplicates for 4 sets of experimental conditions: the photobleaching control, which was kept protected from light; the detergent-only sample, where gels were incubated in Tris-HCl and 2% SDS at RT; the heat-only sample, where gels were incubated in Tris-HCl at 55–57 °C; and the heat and SDS sample, where gels were incubated in Tris-HCl and 2% SDS at 55–57 °C. Gels were incubated in their respective conditions for 1 h. After incubation, all gels (except the photobleaching controls) were washed in 1X TBST for 1 h, rinsed with DI water, dried under a nitrogen stream, and scanned with a laser microarray scanner.

### Protein molecular mass experiments

To determine the impact of molecular mass on protein signal loss, BMPA hydrogels immobilized with either 2.1 μM OVA labeled with Alexa Fluor 488 (OVA488), or 2.1 μM TI labeled with Alexa Fluor 488 (TI488), were created. Each protein species tested had two sets of gels created in triplicates: a photobleaching control, and the treatment group. Gels were fabricated, and measured, as described in the sections above. After fabrication, gels belonging to the treatment group were incubated in stripping buffer at 55–57 °C for 1 h. Gels were then washed in 1X TBST for 1 h, rinsed with DI water, dried under a nitrogen stream, and scanned with a laser microarray scanner.

### Reprobing experiments

To determine the corresponding immunoassay signal loss with reprobing rounds, BMPA gels consisting of immobilized RNase-488 with DOL = 1.23 were fabricated as described above, at an initial in-solution concentration of 125 nM. Six gels were fabricated to create two sets of triplicates: the photobleaching controls and the treatment group. Initially, all gels were immunoprobed, which consisted of a 2 h incubation with a 1:10 dilution of 1 mg/mL primary anti-RNase antibody in 2% BSA/TBST solution, followed by a 1 h wash in TBST, another 1 h incubation with a 1:20 dilution of 2 mg/mL of secondary antibody labeled with Alexa Fluor 633 in 2% BSA/TBST solution, and a final 1 h wash in TBST. Since immunoreagent dilutions were fairly high, we used small volumes of immunoreagent solutions (40 μL per gel) to conserve antibody, and performed immunoprobing by sandwiching antibody solution between the gel and a glass plate. Gels were then rinsed with DI water, and dried under a N_2_ stream. After the wash step, all gels were imaged with a laser microarray scanner. Following the initial round of immunoprobing, photobleaching control gels were kept dry for the duration of the experiment, but scanned with the remaining gels at the end of each stripping and reprobing cycle. Gels from the treatment group were subjected to 1 h of stripping with harsh stripping buffer at 55–57 °C, washed with TBST, and had any residual fluorescence scanned with the Genepix microarray scanner. Following this, the treatment group gels were reprobed with an additional set of antibodies, as previously described. The treatment group was stripped and reprobed in this manner for a total of 6 times.

### Data analysis and quantitation

Fluorescence micrographs of BMPA gels were analyzed using custom analysis scripts written in MATLAB R2018a. To align micrographs from sequential stripping experiments, we utilized the Speeded Up Robust Features (SURF) algorithm from MATLAB’s Image Processing Toolbox. Statistical analysis of summed fluorescence intensity, and signal-to-noise ratios, were performed using R.

## Supplementary information

Supplementary Information

## Data Availability

The datasets generated during and/or analyzed during the current study are available from the corresponding author on reasonable request.

## References

[1] Yu C, Woods A, Levison D (1992). The assessment of cellular proliferation by immunohistochemistry: a review of currently available methods and their applications. Histochemistry.

[2] Campisi J, d’Adda di Fagagna F (2007). Cellular senescence: when bad things happen to good cells. Nat. Rev. Immunol..

[3] Christianson TA (1998). NH2-terminally truncated HER-2/neu protein: relationship with shedding of the extracellular domain and with prognostic factors in breast cancer.. Cancer Res.

[4] Hughes AJ (2014). Single-cell western blotting. Nat. Methods.

[5] Tentori, A. M., Yamauchi, K. A. & Herr, A. E. Detection of Isoforms Differing by a Single Charge Unit in Individual Cells. *Angew. Chemie* **128, **12431–12435 (2016). 10.1002/anie.201606039 PMC5201312 27595864

[6] Yamauchi KA, Herr AE (2017). Subcellular western blotting of single cells. Microsystems Nanoeng..

[7] Xu W, Luikart AM, Sims CE, Allbritton NL (2010). Contact printing of arrayed microstructures. Anal. Bioanal. Chem..

[8] Ornoff DM, Wang Y, Proctor A, Shah AS, Allbritton NL (2016). Co-fabrication of chitosan and epoxy photoresist to form microwell arrays with permeable hydrogel bottoms. Biomaterials.

[9] Tanase CP, Albulescu R, Neagu M (2011). Application of 3D hydrogel microarrays in molecular diagnostics: Advantages and limitations. Expert Rev. Mol. Diagn..

[10] Spencer, A. R. *et al*. Electroconductive Gelatin Methacryloyl-PEDOT:PSS Composite Hydrogels: Design, Synthesis, and Properties. *ACS Biomater. Sci. Eng.* **4,** 1558–1567 (2018). 10.1021/acsbiomaterials.8b00135 PMC11150039 33445313

[11] Bettadapur A (2016). Prolonged Culture of Aligned Skeletal Myotubes on Micromolded Gelatin Hydrogels. Sci. Rep..

[12] Chung K (2013). Structural and molecular interrogation of intact biological systems. Nature.

[13] Chen, F., Tillberg, P. W. & Boyden, E. Expansion Microscopy. *Science* **347** (2015). 10.1126/science.1260088 PMC4312537 25592419

[14] Zhang X, Li L, Luo C (2016). Gel integration for microfluidic applications. Lab Chip.

[15] Kim D, Herr AE (2013). Protein immobilization techniques for microfluidic assays. Biomicrofluidics.

[16] Chozinski TJ (2016). Expansion microscopy with conventional antibodies and fluorescent proteins. Nat. Methods.

[17] Hughes AJ, Herr AE (2012). Microfluidic Western blotting. Proc. Natl. Acad. Sci. USA.

[18] Appleyard DC, Chapin SC, Doyle PS (2011). Multiplexed Protein Quantification with Barcoded Hydrogel Microparticles. Anal. Chem..

[19] Schneider MH, Tran Y, Tabeling P (2011). Benzophenone absorption and diffusion in poly(dimethylsiloxane) and its role in graft photo-polymerization for surface modification. Langmuir.

[20] O’Neill RA (2006). Isoelectric focusing technology quantifies protein signaling in 25 cells. Proc. Natl. Acad. Sci..

[21] Dormán G, Prestwich GD (1994). Benzophenone photophores in biochemistry. Biochemistry.

[22] Banks JM, Harley BAC, Bailey RC (2015). Tunable, Photoreactive Hydrogel System to Probe Synergies between Mechanical and Biomolecular Cues on Adipose-Derived Mesenchymal Stem Cell Differentiation. ACS Biomater. Sci. Eng..

[23] Kapil, M. A., Pan, Y., Duncombe, T. A. & Herr, A. E. Binding Kinetic Rates Measured via Electrophoretic Band Crossing in a Pseudohomogeneous Format. *Anal. Chem*. **88** (2014). 10.1021/ac403829z 24552202

[24] Neira HD, Herr AE (2017). Kinetic Analysis of Enzymes Immobilized in Porous Film Arrays. Anal. Chem..

[25] Dixon AR (2015). Recent developments in multiplexing techniques for immunohistochemistry. Expert Rev. Mol. Diagn..

[26] Tsurui H (2000). Seven-color Fluorescence Imaging of Tissue Samples Based on Fourier Spectroscopy and Singular Value Decomposition. J. Histochem. Cytochem..

[27] Gerdes MJ (2013). Highly multiplexed single-cell analysis of formalin-fixed, paraffin-embedded cancer tissue. Proc. Natl. Acad. Sci. USA.

[28] Vira S, Mekhedov E, Humphrey G, Blank PS (2011). Fluorescent labeled antibodies - balancing functionality and degree of labeling. Anal. Biochem..

[29] Dhawan S (2006). Signal amplification systems in immunoassays: Implications for clinical diagnostics. Expert Rev. Mol. Diagn..

[30] Wählby C, Erlandsson F, Bengtsson E, Zetterberg A (2002). Sequential immunofluorescence staining and image analysis for detection of large numbers of antigens in individual cell nuclei. Cytometry.

[31] Glass G, Papin JA, Mandell JW (2009). SIMPLE: A sequential immunoperoxidase labeling and erasing method. J. Histochem. Cytochem..

[32] Zrazhevskiy P, True LD, Gao X (2013). Multicolor multicycle molecular profiling with quantum dots for single-cell analysis. Nat. Protoc..

[33] Kaufmann SH, Ewing CM, Shaper JH (1987). The erasable Western blot. Anal. Biochem..

[34] Ku T (2016). Multiplexed and scalable super-resolution imaging of three-dimensional protein localization in size-adjustable tissues. Nat. Biotechnol..

[35] Gallagher S, Winston SE, Fuller SA, Hurrell JGR (2008). Immunoblotting and Immunodetection. Curr. Protoc. Cell Biol..

[36] Sinkala, E. *et al*. Profiling protein expression in circulating tumour cells using microfluidic western blotting. *Nat*. *Commun.* **8** (2017). 10.1038/ncomms14622 PMC5376644 28332571

[37] Reynolds JA, Tanford C (1970). Binding of Dodecyl Sulfate to Proteins at High Binding Ratios. Possible Implications for the State of Proteins in Biological Membranes. Proc. Natl. Acad. Sci..

[38] Jocelyn PC (1987). Chemical reduction of disulfides. Methods Enzymol..

[39] Cooper A (2000). Heat capacity of hydrogen-bonded networks: An alternative view of protein folding thermodynamics. Biophys. Chem..

[40] Patyukova E, Rottreau T, Evans R, Topham PD, Greenall MJ (2018). Hydrogen Bonding Aggregation in Acrylamide: Theory and Experiment. Macromolecules.

[41] Lu P, Hsieh Y-L (2009). Organic compatible polyacrylamide hydrogel fibers. Polymer.

[42] Suzawa T, Shirahama H (1991). Adsorption of plasma proteins onto polymer latices. Adv. Colloid Interface Sci..

[43] Saeed IA, Ashraf SS (2009). Denaturation studies reveal significant differences between GFP and blue fluorescent protein. Int. J. Biol. Macromol..

[44] Panchuk-Voloshina N (1999). Alexa Dyes, a Series of New Fluorescent Dyes that Yield Exceptionally Bright, Photostable Conjugates. J. Histochem. Cytochem..

[45] Vlassakis J, Herr AE (2015). Effect of Polymer Hydration State on In-Gel Immunoassays. Anal. Chem..

[46] Bay, H., Tuytelaars, T. & Van Gool, L. SURF: Speeded Up Robust Features. In *Computer Vision - ECCV 2006,* 404–417 (2006).

[47] Albiach, M. R., Guerri, J. & Moreno, P. Albiach, Multiple Use of Blotted Polyvinylidene Difluoride Membranes Immunostained with Nitro Blue Tetrazolium. *Anal*. *Biochem*. 25–28 (1994). 10.1006/abio.1994.1373 7985800

[48] Wolf WJ (1977). Physical and chemical properties of soybean proteins. J. Am. Oil Chem. Soc..

[49] Takeuchi K (2014). Accuracy of Protein Size Estimates Based on Light Scattering Measurements. Open J. Biophys..

[50] Kang C (2016). Single cell resolution western blotting. Nat. Protoc..

[51] Jøssang T, Feder J, Rosenqvist E (1988). Photon correlation spectroscopy of human IgG. J. Protein Chem..

[52] Holmes DL, Stellwagen NC (1991). Estimation of polyacrylamide gel pore size from Ferguson plots of linear DNA fragments. Electrophoresis.

[53] Liu L, Li P, Asher SA (1999). Entropic trapping of macromolecules by mesoscopic periodic voids in a polymer hydrogel. Nat. Lett..

[54] Zustiak SP, Wei Y, Leach JB (2013). Protein-hydrogel interactions in tissue engineering: mechanisms and applications. Tissue Eng. Part B. Rev..

[55] Risbud MV, Bhond RR (2000). Polyacrylamide-Chitosan Hydrogels: *In Vitro* Biocompatibility and Sustained Antibiotic Release Studies. Drug Deliv..

