# Supplementary material for: Multiplexed in-gel microfluidic immunoassays: characterizing protein target loss during reprobing of benzophenone-modified hydrogels
Source: Sci Rep. 2019 Oct 28;9:15389. doi: 10.1038/s41598-019-51849-8 (PMC6817870; doi:10.1038/s41598-019-51849-8)
Supplement: Supplementary file 1 — Supplementary Information [file 41598_2019_51849_MOESM1_ESM.pdf]

Supporting Information

Multiplexed in-gel microfluidic immunoassays: characterizing protein target loss during reprobing of benzophenone-modified hydrogels

Anjali Gopal[†,‡] & Amy E. Herr[†,‡,§]

†Department of Bioengineering and ‡ The UC Berkeley/UCSF Graduate Program in Bioengineering, University of California, Berkeley, Berkeley, California 94720, United States
[§] Chan Zuckerberg BioHub, San Francisco, California, 94158, United States

Supplementary Note: Thermocycling experiments for protein-fluorophore conjugates.

Experimental Information. In order to ensure stability of protein-fluorophore conjugates upon heat denaturation, we performed thermocycling experiments. 1.05 μM concentrations (corresponding to the concentration of protein-conjugates used in our in-gel experiments, after accounting for partitioning effects[1,2]) of each protein-conjugate (OVA, TI, and RNase) was prepared in TBST and split into two lots: the first lot was protected from light and stored at room temperature, whereas the second lot was protected from light and incubated in a 55-57^{0}C water bath for 1 h. After incubation, 5 sets of 100 μL samples were drawn from each lot and measured using a plate reader (Tecan Infinite M200 Pro, Tecan Systems Inc., San Jose, CA). Samples were repeatedly incubated and measured in this manner for a total of four cycles.

Results. Our results indicate that all protein-fluorophore conjugates are stable to thermoycling, as demonstrated by the minimal change in fluorescence intensities between the control and thermocycle conditions (**Supplementary Figure S1)** across multiple incubation cycles**.** These results suggest that if protein conjugates immobilized in BMPA hydrogels demonstrate fluorescence loss greater than their corresponding photobleaching controls, then this fluorescence loss is due to loss of protein-conjugates from the hydrogel. Moreover, since heat denatures both hydrogen bonds and hydrophobic bonds[3], we anticipate that we would observe similar results for "cycling" studies with other components of stripping buffer (e.g., SDS).

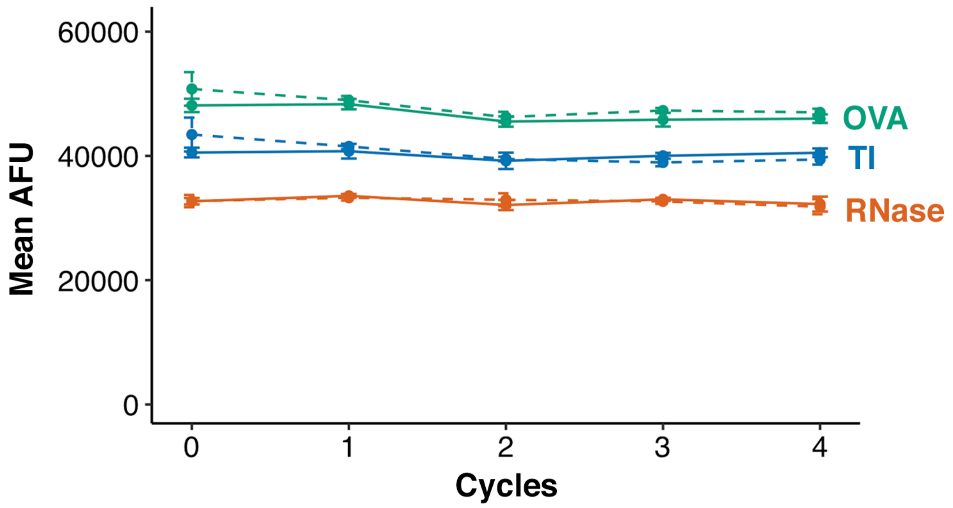

Supplementary Figure S1. Thermocycling experiments demonstrate stability of protein/fluorophore conjugates upon denaturation. 1.05 μM concentrations of OVA, TI, and RNase protein-fluorophore conjugates in TBST were thermocycled for 4 rounds. After each round, fluorescence readings were taken from 100 μL aliquots (n = 5) of each protein-fluorophore conjugate using a plate reader. Dotted lines represent photobleaching controls; solid lines represent samples that were subject to thermocycling. Differences in starting AFU can be attributed to differences in the degree-of-labeling of each protein conjugate.

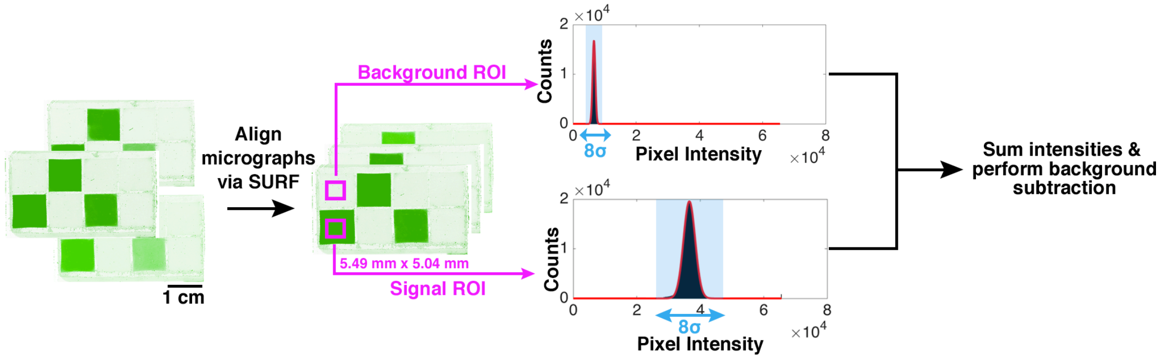

Supplementary Figure S2. Data analysis workflow for stripping experiments using BMPA hydrogels with immobilized proteins. Micrographs from sequential stripping rounds are aligned and compiled into an image stack using the Speeded Up Robust Features ("SURF") function in MATLAB's image processing toolbox. Once stacked, a 5.49 mm x 5.04 mm region-of-interest is identified for every immobilized protein region. The pixel intensities from each ROI are fit to a Gaussian distribution, and any outlier pixels (> 4σ away from the mean) are re-scaled to the median pixel intensity value. These pixel intensities are then summed. A similar procedure is performed for adjacent background ROIs, which is subsequently used for background subtraction.

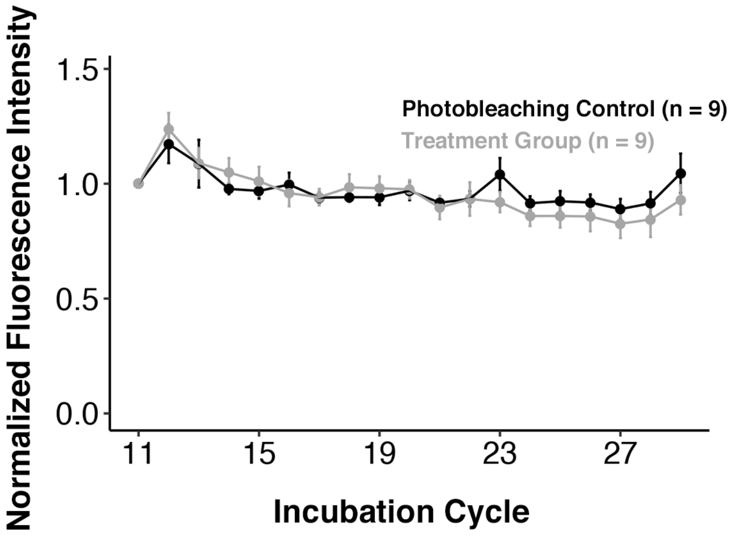

Supplementary Figure S3. Signal loss of immobilized protein target from BMPA hydrogels from rounds 11 – 29. Signal from each ROI was normalized to the ROI signal from round 11. The overlapping traces between the photobleaching control and the treatment group suggests that the majority of signal loss observed in the treatment group after round 11 cannot be isolated from the effects of photobleaching and/or instrument variation. The slight decrease in the treatment group's intensity during round 24 may be due to a 2 month gap between scanning round 23 and round 24; the time delay between the majority of other successive incubation cycles was < 1 week.

Supplementary Table S1: P-Values for Mann-Whitney U-Tests Between Photobleaching Control and Treatment Group for Rounds 11 - 29

Round	P-Value	Group with Higher Mean Signal
11	N/A	N/A
12	0.190	Treatment
13	0.931	Treatment
14	0.008	Treatment
15	0.019	Treatment
16	0.258	Control
17	1.000	Treatment
18	0.094	Treatment
19	0.113	Treatment
20	0.931	Treatment
21	0.340	Control
22	1.000	Control
23	0.002	Control
24	0.011	Control
25	0.011	Control
26	0.011	Control
27	0.019	Control
28	0.136	Control
29	0.003	Control

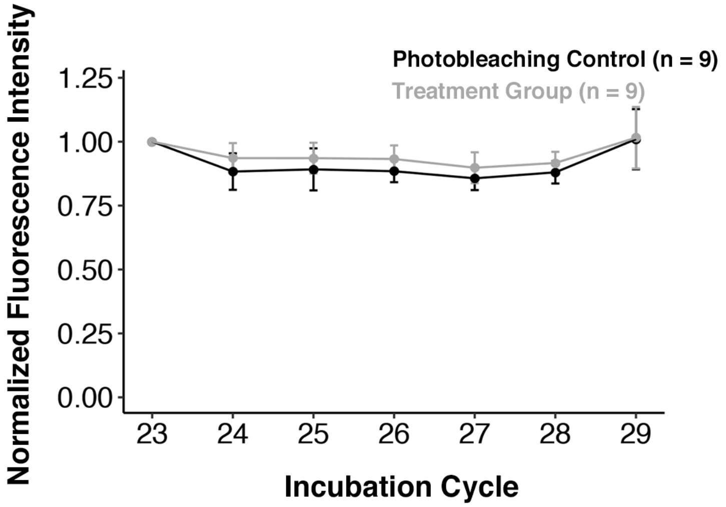

Supplementary Figure S4. Signal loss of immobilized protein target from BMPA hydrogels from rounds 23 – 29. Signal from each ROI was normalized to the ROI signal from round 23. Note that the photobleaching control traces have more signal loss than the treatment group, suggesting that signal loss observed in the treatment group cannot be isolated from the effects of photobleaching and/or instrument variation.

Supplementary Table S2: P-Values for Mann-Whitney U-Tests Between Photobleaching Control and Treatment Group for Rounds 23 - 29

Round	P-Value	Group with Higher Mean Signal
**23**	N/A	N/A
**24**	0.113	Treatment
**25**	0.258	Treatment
**26**	0.031	Treatment
**27**	0.113	Treatment
**28**	0.113	Treatment
**29**	1.000	Treatment

Supplementary Note: Spatial Non-Uniform Probing in Treatment Group
Although we initially fabricated 3 gels to assess immunoassay signal loss after multiple rounds of stripping and reprobing, we observed significant spatial non-uniformity in one of our three gels, which increases the variance in the measurements of immunoassay signal loss after multiple reprobing rounds (see **Supplementary Figure S5**).

A micrograph of probing non-uniformity in Gel 2 can be seen in **Supplementary Figure S6**. Furthermore, as evident in **Supplementary Table S3**, Gel 2, Region 2 has significant increase in normalized immunoprobed fluorescence between Round 0 and Round 1. In general, immunoprobed fluorescence signal should not increase between Round 0 and Round 1, since we have demonstrated significant protein loss between those two rounds (see **Figure 6a**). We attribute this anomalous immunoprobing fluorescence increase to non-uniform probing of Gel 2 during its initial probing round.

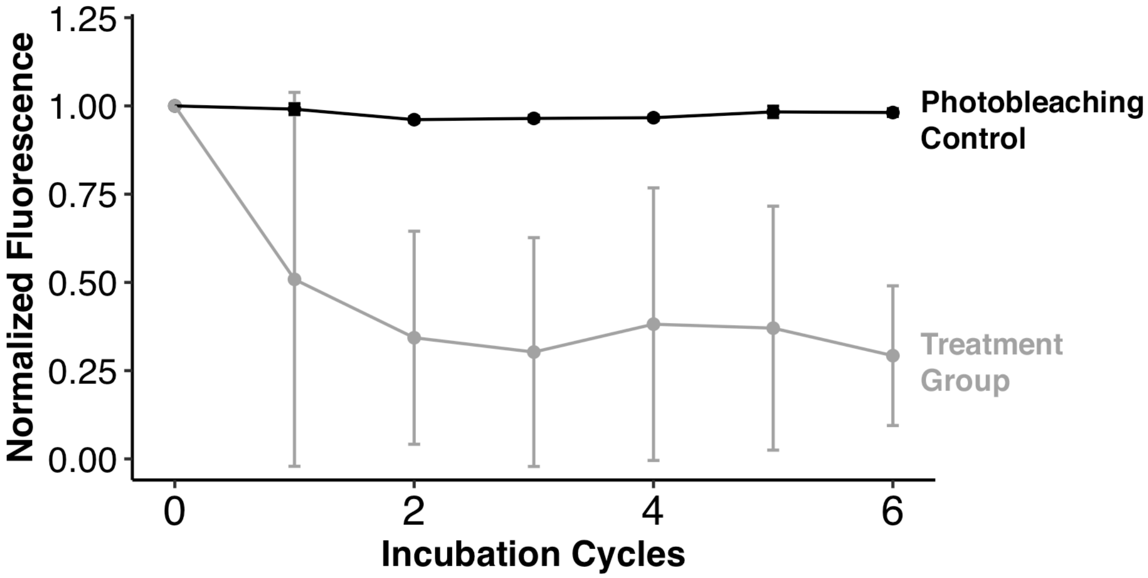

Supplementary Figure S5. Fluorescence Loss of Immunoreagents during Serial Stripping and Reprobing Cycles with Three Hydrogels. Fluorescence loss of immunoreagents in BMPA hydrogels immobilized with RNase-488 and immunoprobed with primary (Rb anti-RNase) and fluorescent secondary (Gt anti-Rb conjugated to Alexa Fluor 633) antibodies. Each incubation cycle following round 0 corresponds to one round of stripping, followed by immediate reprobing with new immunoreagents. The large variation in the treatment group is due to non-uniform probing conditions from Gel 2 (see Supplementary Figure S6).

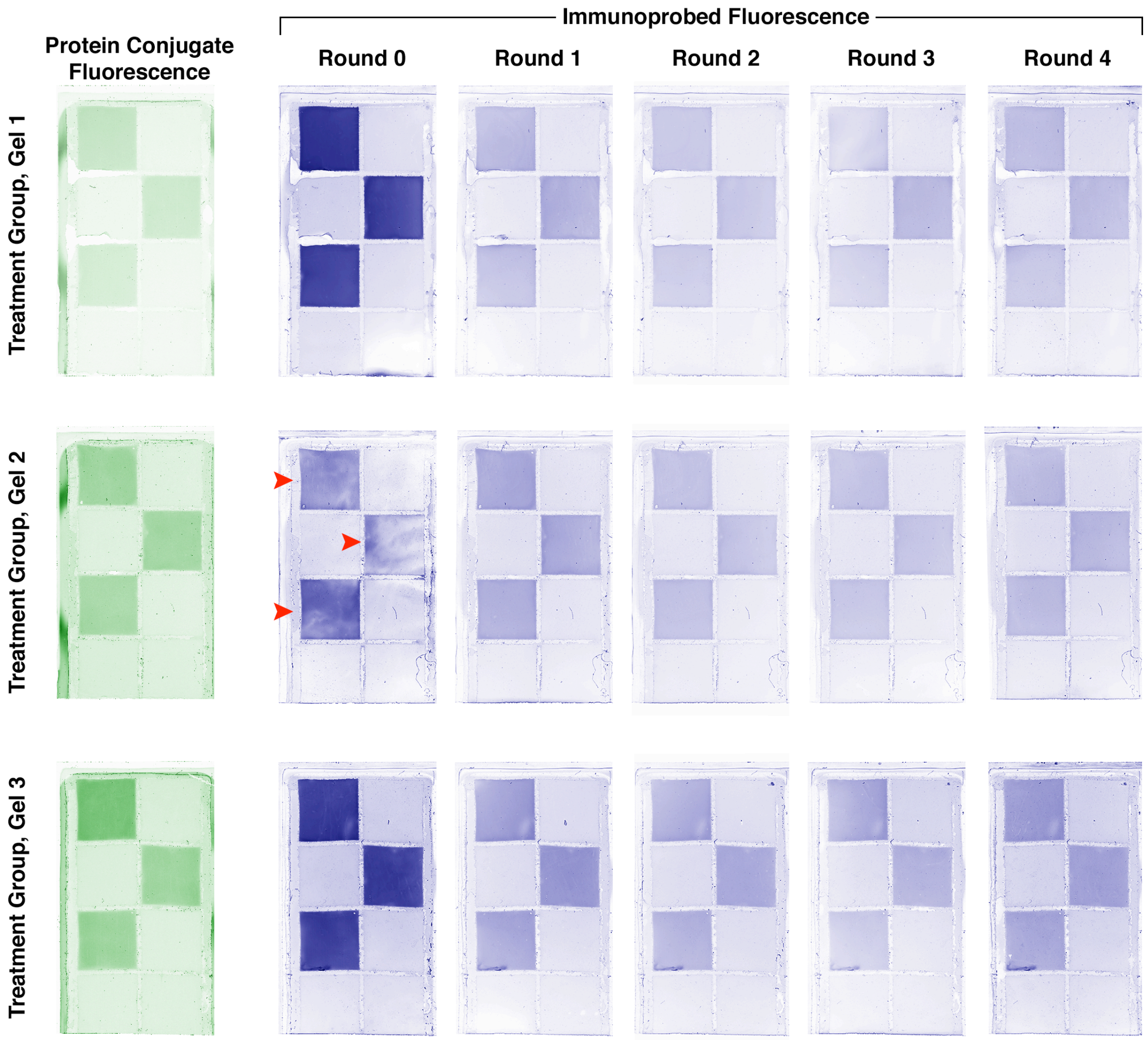

Supplementary Figure S6. False-color micrographs of BMPA hydrogels immobilized with RNase-488 and immunoprobed with antibody. The green micrographs depict protein-conjugate fluorescence (RNase-488), whereas the blue micrographs represent immunoprobed signal after every round of stripping and reprobing. The initial immunoprobing round (Round 0) for gel 2 had large non-uniformities, which have been represented by the red arrows. All other micrographs, including micrographs obtained from further reprobing of gel 2, are uniform.

Supplementary Table S3: Normalized Immunoprobed Fluorescence Values after Reprobing Rounds

Spot ID	Round 0	Round 1	Round 2	Round 3	Round 4	Round 5	Round 6
**Gel 1 – Region 1**	1.000	0.173	0.137	0.044	0.140	0.201	0.223
**Gel 1 – Region 2**	1.000	0.295	0.193	0.261	0.216	0.173	0.133
**Gel 1 – Region 3**	1.000	0.181	0.156	0.102	0.149	0.198	0.173
**Gel 2 – Region 1**	1.000	**0.692**	0.466	0.402	0.426	0.529	0.423
**Gel 2 – Region 2**	1.000	**1.857**	1.102	1.118	1.384	1.234	0.754
**Gel 2 – Region 3**	1.000	**0.434**	0.331	0.272	0.321	0.373	0.352
**Gel 3 – Region 1**	1.000	0.291	0.228	0.187	0.289	0.194	0.189
**Gel 3 – Region 2**	1.000	0.391	0.269	0.230	0.273	0.273	0.240
**Gel 3 – Region 3**	1.000	0.263	0.208	0.108	0.237	0.156	0.143

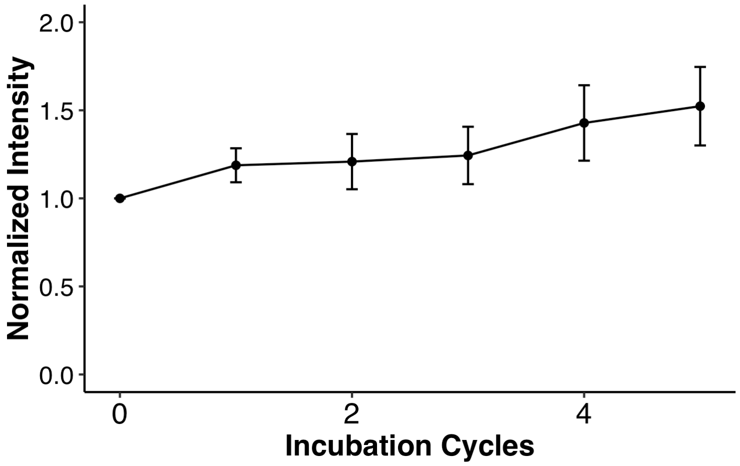

Supplementary Figure S7. Normalized intensity of residual immunoprobing fluorescence after stripping rounds. Immunoprobed gels that were stripped were scanned immediately after stripping but before reprobing (n = 6). Residual signal is attributable to immunoreagents that were not fully removed from the gel.

References

1. Hughes, A. J. *et al.* Single-cell western blotting. *Nat. Methods* **11**, 749–55 (2014).
2. Vlassakis, J. & Herr, A. E. Effect of Polymer Hydration State on In-Gel Immunoassays. *Anal. Chem.* **87**, 11030-11038 (2015).
3. Cooper, A. Heat capacity of hydrogen-bonded networks: An alternative view of protein folding thermodynamics. *Biophys. Chem.* **85**, 25–39 (2000).
